# Microvascular Dynamics and Hemodialysis Response of Patients With End-Stage Renal Disease 

**DOI:** 10.3389/fbioe.2022.836990

**Published:** 2022-05-18

**Authors:** Jen-Shih Lee, Lian-Pin Lee

**Affiliations:** ^1^ Department of Biomedical Engineering, University of Virginia, Charlottesville, VA, United States; ^2^ Global Monitors, Inc., San Diego, CA, United States

**Keywords:** blood volume, microcirculation, plasma protein concentration, hematocrit, hemodialysis

## Abstract

In our previous analysis of three sets of hemodialysis studies, we found that patients possessing higher hematocrit have a higher filtration coefficient KSo and more fluid being restituted from the tissue. A new dynamic analysis is developed to reveal how the plasma protein concentration, restitution volume, and plasma volume are changing over the time course of 240 min hemodialysis. For patients with the filtration coefficient KSo as 0.43 or 5.88 ml/min/mmHg, we find that the restitution rate would reach 50% of the extraction rate in 5.3 or 57.4 min, respectively. By the end of hemodialysis, the restitution rate of both patients asymptotically approaches a value of 0.93 ml/min which is slightly higher than the extraction rate of 9.03 ml/min. The plasma volume drops by 10% of the total plasma volume in 11 min for patients with low KSo and drops by 2.1% and turns around to an increasing trend in 5.6 min for patients with high KSo. These results suggest that the filtration coefficient acts like a facilitator in restituting more fluid from the tissue to compensate for the loss of plasma volume due to extraction. The hematocrit data of three sets of hemodialysis also indicate that significant microvascular blood volume is shifted from small veins toward the venous side of macrocirculation. A better understanding of how the factors examined here cause hypovolemia can be the basis for one to modify the hemodialysis process such that the development of hypovolemia can be avoided over the course of hemodialysis.

## 1 Introduction

Over the course of hemodialysis (HD), the continuous extraction of ultrafiltrate from the blood by the dialyzer will increase the plasma protein concentration (PPC) from Cp to Cp’. For three groups of patients with normal, anemic, and more anemic hematocrit, the PPC increment (Cp’-Cp)/Cp’ is found as 11.7, 10.2, and 7.9%, respectively (Schneditz et al., 1992; Minutolo et al., 2003). The full circulation analysis (FCA) of these end-point protein data reveals that the large drop in the PPC increment is mainly due to the patients having these arterial hematocrits Ha (42.4 ± 3.5%, 33.5 ± 2.4%, and 27.9 ± 3.1%, respectively) have these filtration coefficients KSo (0.43, 0.85, and 5.88 ml/min/mmHg, respectively, personal communication). A larger filtration coefficient is also shown by the FCA to associate with more fluid being restituted from the tissue and lesser reduction in the total plasma volume for patients with lower hematocrit. This impact of the filtration coefficient on plasma volume reduction suggests that the filtration coefficient acts like a factor facilitating the prevention of hypovolemia. In this article, we will expand the end-point analysis to one that can predict how these variables, namely, PPC, restitution volume, plasma volume, microvascular blood pressure, and rate of the restitution are changing over the course of HD. A total of three sets of computations are made for patients having the three filtration coefficients mentioned previously. By comparing the temporal changes of the variables so calculated will help us better understand how and why that the filtration coefficient can be a facilitating factor for the patient to generate a fluid restitution to adequately compensate for the fluid extracted by the dialyzer and hence the prevention of the development of hypovolemia.

In the FCA, the full circulation is divided into three compartments: the macrocirculation, microcirculation, and the splenic microcirculation. The blood flowing in six groups of microvessels (the small arteries, arterioles, capillaries, post-capillaries, venules, and small veins) has a microvessel hematocrit in the range of 90–20% of Ha (Lipowsky et al., 1980). The blood pressure in the small arteries can be 100 mmHg, while that in the small veins is 2–4 mmHg. The organs in our body may have different filtration coefficients. In the FCA, the six groups are grouped into one microcirculation compartment with Pmic being its blood pressure, Hmic as its microvascular hematocrit of the microcirculation, and KSo as its filtration coefficient. Because the red blood cells (RBCs) in the splenic microcirculation are tethered by the microvessel wall, the hematocrit there can be 2.07 times of Ha (Gibson et al., 1946). Equations are formulated in our previous study to describe the transvascular fluid and protein movement between the microcirculation and the tissue. The equations are used to deduce from the measured PPC increment (Cp’-Cp’)/Cp’ and hematocrit increment (Ha’-Ha)/Ha’, the filtration coefficient KSo, the change in plasma volume ΔVp, and the reduction in microvascular blood volume ΔVmic. We will use the morphometric and hemodynamic characteristics of the microcirculation to deduce a value for Pmic and Hmic and to partition the calculated microvascular blood volume reduction to the six microvessel groups. The FCA simulates the transvascular protein movement as the transport of a fluid across the endothelia having γCp as its protein concentration. The constant γ is termed as the permeability fraction. A way to find the value of γ is described.

A hematocrit equation has been established in FCA to show how these three factors, namely, a change in plasma volume (ΔVp), a change in microvascular blood volume (ΔVmic), and a splenic RBC release change of the arterial hematocrit. Graphical results are generated to show how these three factors alter the arterial hematocrit over the course of HD. With known ΔVmic, we can calculate the change in macrovascular blood volume. The meaning of a change in microvascular blood volume and the impact of a change in macrovascular blood volume on cardiac filling are elaborated from the perspective of a 13-generation circulation model (Rothe 2011).

For many hypovolemia studies, the increase in hematocrit over the course of HD is used to determine the reduction in plasma volume over the course of HD (Schneditz et al., 1992; Calvacanti et al., 2006; Dasselaar et al., 2007; Booth et al., 2011). The Van Beaumont (1972) hematocrit equation (VBHE) used in those studies can be derived from the hematocrit equation of the FCA under the assumption of no microvascular volume change and no splenic RBC release. In the study carried out by Schneditz et al. (1992), the blood volume change calculated from VBHE is used in their numerical procedure to determine the filtration coefficient of the more anemic patients. In FCA, we use the PPC data and the protein equation to determine the reduction in plasma volume and the filtration coefficient. The differences that are generated by these two computation procedures are examined.

## 2 Analyses, Results, and Discussions

*The process on changing plasma protein concentration.* In this part of FCA, the plasma space in the entire circulation is treated as one compartment. Before HD, there is a constant filtration flux Jf (0) flowing out of the plasma compartment to the tissue. The 0 in the parenthesis identifies the quantity as one at the initiation of HD (*t* = 0). This flux carries a protein concentration Cf. Meanwhile, there is a constant lymphatic return Qlym which carries a protein concentration Clym. As the plasma volume and the PPC are not likely to change before the initiation of HD, we have the following requirements:

Qlym=Jf(0)  and  Qlym Clym=Jf(0)Cf (1)

As the interstitial space is being flushed by the filtration, these two equalities also indicate that the protein concentration in the interstitial fluid space Ct equals Cf. We regard that the transvascular fluid movement Jf(t) is governed by the Starling hypothesis, that is,

Jf(t)=KSo{Pmic(t)−Pt−σ[πp(t)−πt]} (2)

where KSo is the filtration coefficient, Pmic (0) is a representative hydrostatic pressure of the microcirculation, Pt is the hydrostatic pressure on the tissue side, σ is the reflection coefficient, πp(t) is the plasma colloidal osmotic pressure (COP) at time t, and πt is the tissue COP. Over the course of HD, the total volume of the fluid restituted from the tissue is given by:

$$\Delta \mathrm{Vr}=\int_{0}^{\Delta T}\mathrm{KSo}\left[\sigma \left(\pi p\left(t\right)-\pi t\right)-\left(\mathrm{Pmic}\left(t\right)-\mathrm{Pt}\right)\right]dt+\Delta T\cdot \text{Qlym}\;$$ (3)

We consider that the volume of fluid being restituted from the tissue to the circulation is much smaller than the fluid volume of the interstitial fluid space. Thus, we can assume that the interstitial fluid pressure and the tissue COP are not altered by HD. Thus, the replacement of Qlym in Eq. 3 by Jf (0) converts Eq. 3 to the following form:

$$\Delta \mathrm{Vr}=\int_{0}^{\Delta T}\mathrm{KSo}\left\{\sigma \left[\pi p\left(t\right)-\pi p\left(0\right)\right]-\left[\mathrm{Pmic}\left(t\right)-\mathrm{Pmic}\left(0\right)\right]\right\}dt\;$$ (4)

As derived later, Eq. 4 can be converted to the following algebraic equation:

ΔVr=k KSo[σΔπp−ΔPmic] (5)

where Δπp is πp’-πp, ΔPmic is Pmic’-Pmic, and k is an integration constant (Eq. 8). From now on, quantity without ’ is identified as one at *t* = 0, quantity with ’ as one at *t* = ΔT, and that with (t) as one at time t. Later on, Pmic is identified as a surface average of the blood pressure over the entire surface area of the microcirculation, and the pressure change ΔPmic can be calculated as:

ΔPmic=ζΔVb/Vb (6)

where ζ is termed as the autonomous constant, and ΔVb is Vb’-Vb.

The volume of restitution subtracted by the extraction volume ΔVe is the change in plasma volume:

ΔVp≡Vp′−Vp=ΔVr−ΔVe (7)

As we regard that the splenic RBC release does not change the blood volume of the splenic microvasculature, the change in total blood volume ΔVb is given by:

ΔVb=ΔVp (8)

Let Cr be the protein concentration of the restituted fluid. Then, the protein mass added to the plasma compartment is CrΔVr. Thus, the increase in the protein mass over the course of HD is given by:

Cp′ Vp′=Cp Vp+CrΔVr (9a)

Eqs 7 and 9a can be combined to form the following equation:

ΔCp/Cp′=ΔVe/Vp−(ΔVr/Vp)(1−Cr/Cp′) (9b)

where ΔCp is Cp’-Cp. As described later, we use the permeability fraction γ to characterize Cr as a fraction of Cp:

Cr=γCp (10)

Termed the protein equation, Eq. 9b uses the change in PPC to calculate ΔVr/Vp. The first term ∆Cp/Cp’ in Eq. 9b is termed as the PPC increment (the increase in PPC induced by HD and then normalized by Cp’), the second term ∆Ve/Vp is termed as the extraction increment (the increment in PPC induced by the extraction), and the third term is the restitution dilution (the dilution of PPC by fluid restitution). As Cr is smaller than Cp, the value of restitution dilution is negative. The negativity makes us to name this third term as dilution.

Overall, we have six algebraic equations (Eqs. 5–10) to characterize the transvascular fluid and protein movements. The equations contain four modeling constants: KSo, σ, γ, and ζ. Here, KSo is treated as an input parameter, while σ is taken as 1, γ as 0.09, and ζ as 36.67 mmHg. The hemodialysis process is defined by two parameters: ΔT and ΔVe. Once the initial values of plasma volume Vp, blood volume Vb, and PPC Cp are given, then we can use the equations to solve for these six end-values: Cp’ or ΔCp, Cr, Vp’ or ΔVp, Vb’ or ΔVb, ΔVr, and ΔPmic. The value of Δπp is derived from Cp and Cp’ through the Landis and Pappenheimer (1963) equation (Eq. A6).

The two HD studies carried out by Minutolo et al. (2003) on patients with normal and anemic (N and A) hematocrit and the high ultrafiltration experiment carried out by Schneditz et al. (1992) on patients with more anemic (MA) hematocrit are to be identified as N78%, A72%, and MA18%20 HD, respectively. The percentage defines the ΔVe/Vp imposed to the HD. The first two HDs have 240 min as ΔT. The two digits 20 in the last HD is used to highlight that its HD time ΔT is 20 min. These HD parameters, ΔVe, ΔT, and the initial value of Cp, Vp, and Vb and the final PPC Cp’, can be found or derived from the publications. In our previous analysis, we used Eqs 5–10 and Eq. A6 to calculate these seven variables: Cr, ΔVr, ∆Vp, ∆Vb, Δπp, ΔPmic, and kKSo. Here, we use the equations in the Appendix to calculate the temporal changes of Vr(t), Vp(t), Vb(t), Pmic (t), πp(t), and the integration constant k and filtration coefficient KSo.

The second series of HDs will be identified as low KSo, medium KSo, and high KSo HD. They are to have these filtration coefficients: 0.43, 0.85, and 5.88 ml/min/mmHg, which are the filtration coefficients found for N78%, A72%, and MA18%20 HD, respectively. The initial conditions and the settings of ΔVe and ΔT for this second series are those of N78% HD.

*Microvascular morphometry, hemodynamics, and hemodialysis*. In formulating FCA, two new modeling constants, the permeability fraction γ and the autonomous constant ζ, and the microvascular blood pressure are introduced. In this section, a procedure based on the morphometry and hemodynamics of the microcirculation is presented to assess what likely values are to be set for the two constants and one pressure variable.

**FIGURE 1:**
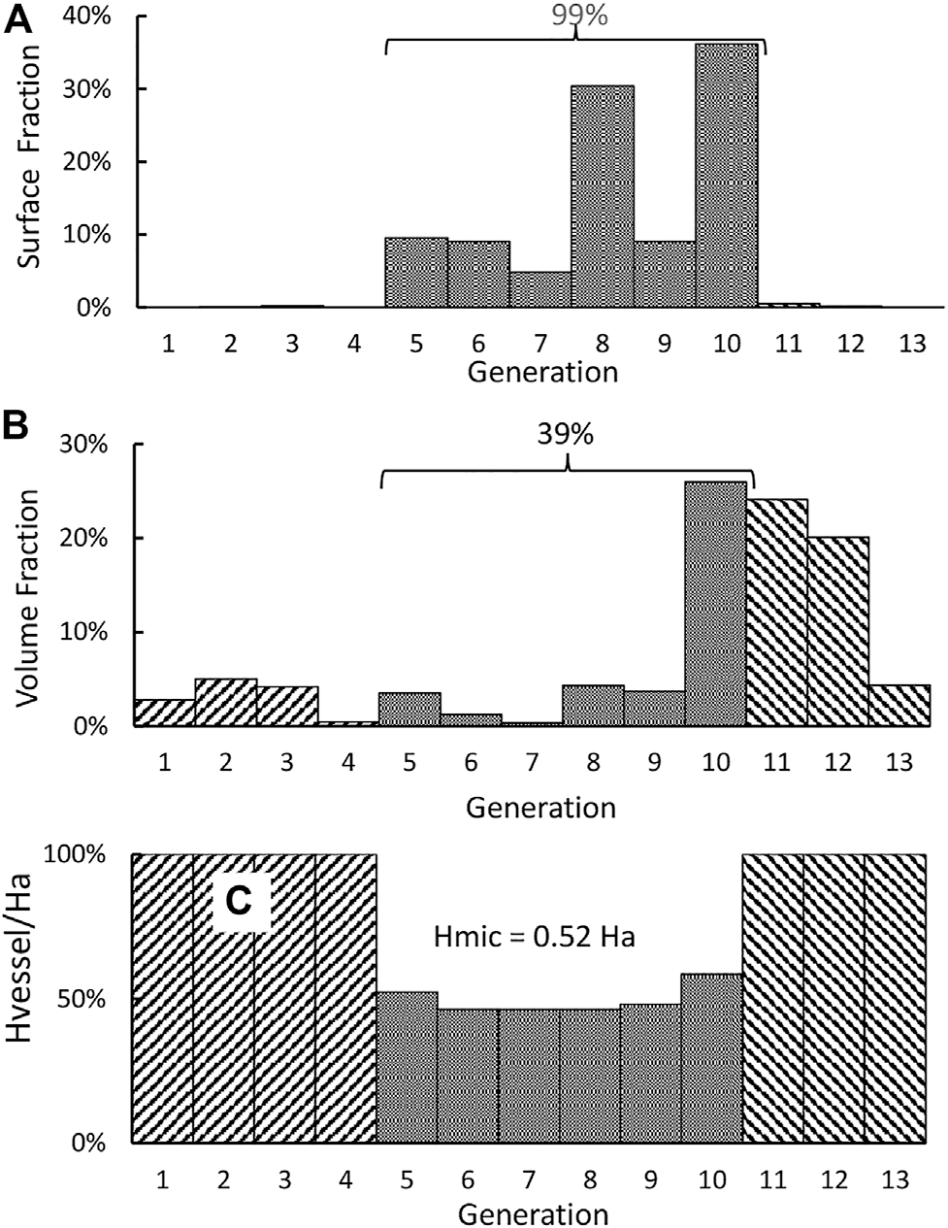
**(A)** Distribution of the surface fraction of a 13-generation central vascular tree. **(B)** Distribution of the volume fraction. **(C)** Distribution of the hematocrit ratio Hvessel/Ha.

The morphometry data (generation number, vessel type, number of vessels, diameter, and length) of a central vascular tree model are reproduced in the first five columns of Table 1 (Rothe 2011). The surface areas and volumes of these vessels are listed in the 5th and 7th column of the table. With So and Vo being the total surface area and the total blood volume of the vascular tree, the surface and volume fractions (Sn/So and Vn/Vo) of the 13-generations circulation are depicted in Figures 1A,B.

**TABLE 1:** Morphometry and hemodynamics of a central vascular tree^a^ (Rothe 2011).

*n*	Vessel type	Number N_n_	Diameter D_n_, mm	Length L_n_, mm	Surface area S_n_, m^2^	S-axis (%)	Volume V_n_, mL	Ht_n_/Ha	R_n_
1	Aorta	1	10	400	0.01	0.00	31.4	1	2.8
2	Large arteries	40	3	200	0.08	0.01	56.5	1	4.3
3	Main arterial branches	600	1	100	0.19	0.10	47.1	1	11.5
4	Terminal branches	1800	0.6	10	0.03	0.32	5.1	1	3.0
5	Small arteries	4.0E+07	0.019	3.5	8.36	0.35	39.7	0.52	25.1
6	Arterioles	4.0E+08	0.007	0.9	7.92	9.90	13.9	0.46	32.5
7	Capillaries	1.8E+09	0.0037	0.2	4.18	18.95	3.9	0.46	20.6
8	Post-capillary Venules	5.8E+09	0.0073	0.2	26.60	23.73	48.6	0.46	0.4
9	Venules	1.2E+09	0.021	0.1	7.92	54.12	41.6	0.48	0.0
10	Small veins	8.0E+07	0.037	3.4	31.62	63.17	292.5	0.59	0.9
11	Main venule branches	600	2.4	100	0.45	99.29	271.4	1	0.3
12	Large veins	40	6	200	0.15	99.81	226.2	1	0.3
13	Vena cava	1	12.5	400	0.02	99.98	49.1	1	1.1
	Total				87.5	100	1127		101.7

a By including the blood volume in the lungs and heart chambers, the total blood volume is 1376 ml. The cardiac output generating the pre-HD pressure is 1.376 L/min. The unit of resistance R_n_ is mmHg/(L/min).

Through the relation between the *in vivo* microvascular hematocrit and vessel diameter (Lipowsky et al., 1980), we use the diameter reported in the table to set a value as the microvessel hematocrit Hmic,n of the *n*th generation and present it through this ratio α_n_ (= Hmic,n/Ha) in the 9th column of Table 1. For the current investigations, the vessels of the 5th to 10th generation are categorized as microvessels, as their hematocrit ratios are all smaller than unity. Among these six generations, only the capillaries may be considered as rigid (Fung 1966), while other larger microvessels are distensible.

It is noted from Figure 1A that almost 99% of the surface area of the circulation resides in the microcirculation. All blood vessels are lined by endothelial cells, and the filtration and permeation characteristics of various microvessels may be similar to each other. Thus, the total surface area of the microcirculation should be taken as the So in Eq. 2. For this surface distribution, we form an S-axis (the 7th column) to mark the location of a point within the vascular network. As an example, the entrance of the microcirculation (i.e., the entrance of small arteries) is located at an S of 0.35% which is the sum of the surface areas of all vessel upstream of the entrance divided by the total surface area (87.5 m^2^). Correspondently, the exit of the microcirculation is located at an S of 99.3%. The blood volumes tabulated in the 8th column indicate that 70% of the total blood volume resides in the 10th to 12th generation. We calculate the relative viscosity of blood μ* as 13.2 α_n_^2^ + 1.30 α_n_ +1.45 and then take the resistance of the *n*th generation as A∙μ* L_n_/(N_n_∙D_n_^4^). The resistance for each generation so calculated is shown in the last column of Table 1.

**FIGURE 2:**
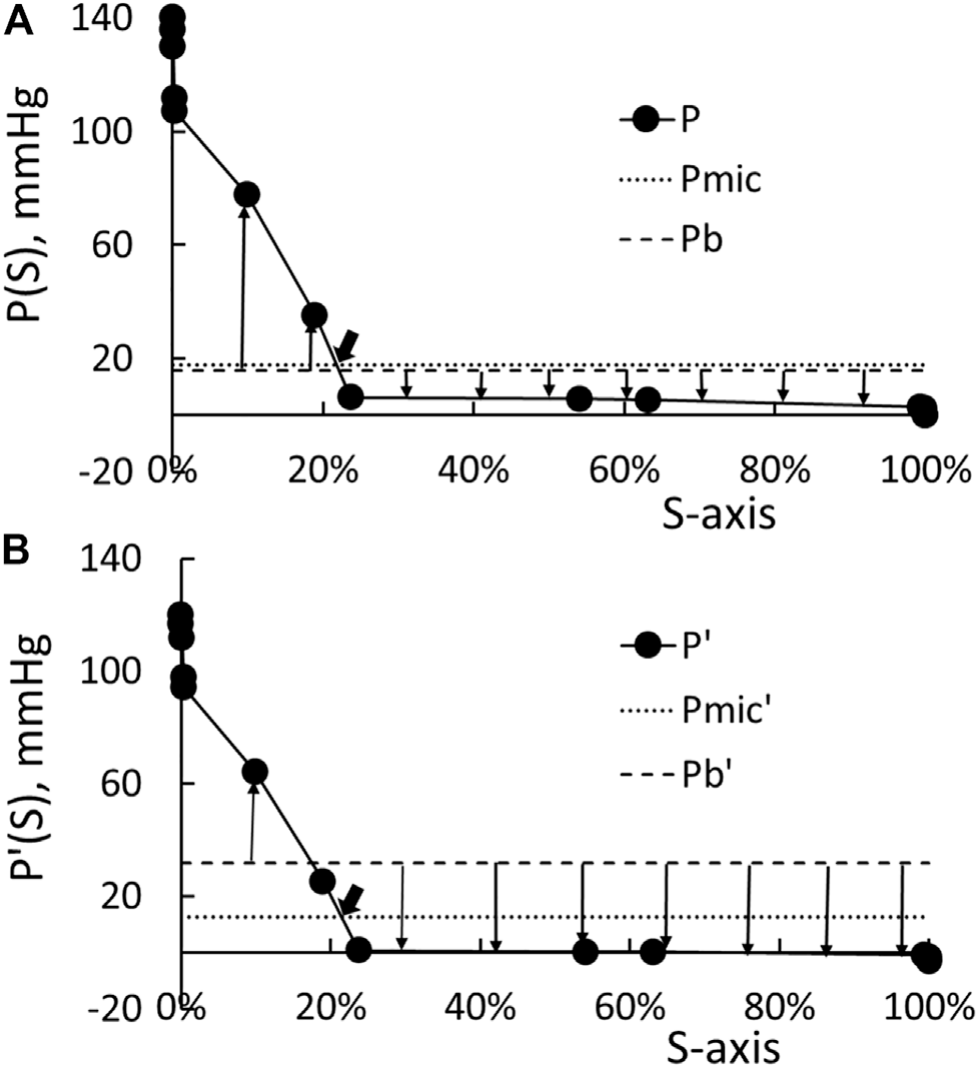
**(A)** Distribution of the pre-HD blood pressure P. Pmic is the surface weighted average of the pressure in the microcirculation, and Pb is the base pressure (Eq. 7). The up arrows indicate fluid filtration to the tissue, and the down arrow indicates fluid absorption by the circulation. **(B)** Distribution of post-HD blood pressure and the correspondent Pmic’ and Pb.

In the next five paragraphs, we will use the data in Table 1 to determine what value should be set for ΔPmic in Eq. 5. Suppose the cardiac output for the central circulation of Table 1 is set at 1.38 L/min, the arterial blood pressure at 140 mmHg, and the venous blood pressure at 0, then we can find a value for A to generate the distribution of blood pressure (the solid line) shown in Figure 2A. This distribution of blood pressure along the S-axis will be taken as that of the patient whose HD has been just initiated.

Based on the studies of Minutolo et al. (2003) and LaForte et al. (1994), the drop in the arterial blood pressure at the end of HD ΔPa is 20 mmHg while the drop in venous blood pressure ΔPv is −3 mmHg. By guessing the cardiac output as 1.21 L/min, setting Pv’ as −3 mmHg, and using the resistance distribution shown in Table 1 to do the pressure calculation, the distribution of blood pressure at the end of HD is shown as the solid line in Figure 2B. The pressure distribution shown in Figure 2A indicates that the pressure in the microcirculation to drop from 110 to 4 mmHg at the beginning of HD, while Figure 2B indicate that the drop is from 94 to 0.2 mmHg at the end of HD.

Let Pb be σ πp (0) + Pt - σ πt. These four pressures Pb, πp, Pt, and πt may be considered as constants as one moves along from the arterial end to the venous end of the microcirculation. The fluid flux Jf in Eq. 2 generated over the microvascular surface at time 0 is now calculated as:

$$\mathrm{Jf}=\int_{0}^{1}\mathrm{KSo}\left(P-\mathrm{Pb}\right)\mathrm{dS}=\mathrm{KSo}\left(\mathrm{Pmic}-\mathrm{Pb}\right)$$ (11)

If KSo is a constant, then the last equality of Eq. 11 shows that Pmic is a surface average of the blood pressure in the microcirculation. The integration of the beginning pressure P shown in Figure 2A yields 19.2 mmHg as the value of Pmic. It is noted that Pb is used in Eq. 11 to show that P-Pb is the net pressure driving the fluid flux Jf. The presence of Pb in Eq. 11 has no effect on the determination of Pmic. With Pmic so determined, we plot the line P = Pmic as the dotted line in Figure 2A. The intercept between the dotted line and solid line as pointed by an arrow in Figure 2A corresponds to an S of 0.22, a location that is slightly downstream of the entrances of capillaries. We choose Pb as 17.2 mmHg so that there is a net driving pressure of 2 mmHg to produce the filtration flux before the initiation of HD (Patients with anemic hematocrit and a body weight of 54 Kg has the KS product as 0.85 ml/min/mmHg. This 2-mmHg pressure is chosen such that the fluid flows through the interstitial corresponding to a lymphatic flow of 1.7 L/day (= 2 mmHg ∙ 0.85 ml/min/mmHg)). The line P = Pb is plotted as the broken line in Figure 2A. Then, the up arrows in Figure 2A originated from the broken line represent filtration for that portion of microcirculation, while the down arrows represent absorption.

We can add the resistances up to form a R-axis like the formation of the S-axis. Then, the plot of the blood pressure P against the R-axis will be a straight line with a negative slope. Let the total resistance be Rt. The intercept pointed at by the big arrow in Figure 2A has f Rt as its resistance with the resistance fraction f taking 0.9 as its value. This is to say that the Pmic can be calculated as:

Pmic=(1−f)Pa+fPv (12)

The computation of the area over the part of blood pressure covered by the up arrows yields the filtration flux Qf as one driven by a pressure differential of 11.3 mmHg (i.e., the flux is 11.3 mmHg KSo), while the absorption flux Qa as one driven by −9.3 mmHg. The sum of the filtration and absorption flux is the net flux which is driven by 2 mmHg.

By the end of HD, the arterial blood pressure drops by about 20 mmHg while the venous blood pressure by 3 mmHg (LaForte et al., 1994; Minutolo et al., 2003). We then guess a cardiac output, set the venous pressure at –3 mmHg, and use the resistances listed in Table 1 to calculate the pressure distribution such that the calculated arterial blood pressure will now be 120 mmHg. The distribution of P′ so calculated is shown as the solid line in Figure 2B. The microvascular blood pressure Pmic’ now takes 14 mmHg as its value. It is located at an S′ of 0.215 and its correspondent resistance fraction f’ takes 0.88 as its value. Because these S′ and f’ closely approximate the S and f derived from the blood pressure before the HD, we use the following formula to determine the change in microvascular blood pressure ΔPmic:

ΔPmic=(1−f)ΔPa+fΔPv (13)

In the CHRE of LaForte et al. (1994), they found that the drops in arterial and venous blood pressure are linearly related to the reduction in blood volume. The substitution of the linear relations to Eq. 13 yields Eq. 6 given earlier. The value of ζ is calculated as 36.7 mmHg for conscious rabbits and 23.7 mmHg for anesthetized rabbits. Because of the dependence on the consciousness of the rabbits, we term ζ as an autonomous constant.

In the following three paragraphs, we describe how ΔVr, Cr, and γ are related to the transvascular fluid and protein movement and what value should be used as γ. For patients with normal hematocrit, the HD induces the COP to increase by 16 mmHg (= Δπp). The new base pressure Pb’ (= Pt + πp’ - πt = Pb + Δπp) is now set as 33.2 mmHg (= 17.2 mmHg +16 mmHg). The line P’ = Pmic’ and P’ = Pb’ are plotted as the dotted and broken line in Figure 2B, respectively. As one sees the filtration arrows in Figure 2B are shorter than those depicted in Figure 2A, while the absorption arrows in Figure 2B are longer than those in Figure 2A. The pressure producing the filtration flux Qf’, absorption flux Qa’, and net flux (= Qf’–Qa’) are driven by these pressure differences 6.0, −25.2, and −19.2 mmHg, respectively.

On the arterial side of the microcirculation, the fluid in the vascular side is being filtrated at the rate Qf(t) from the circulation to the tissue. Let the protein concentration of the filtrated fluid be Cr,f. The semi-permeability of the endothelial lining may only allow a fraction of the protein in the plasma to filtrate to the tissue, i.e. Cr,f will be smaller than Cp. On the venous side, the fluid in the tissue is being absorbed to the circulation at a rate of Qa. Let the protein concentration of the interstitial fluid be Ct and that of the absorbed or restituted fluid be Cr,a which is a fraction of Ct. The integration of these fluid and protein fluxes over the HD time yields the following two integrals:

$$\Delta \text{Vr}=\int_{0}^{\Delta T}\left[\text{Qa}\left(t\right)-\text{Qf}\left(t\right)\right]\text{dt}$$ (14)

$$\Delta \mathrm{Vr}\mathrm{Cr}=\int_{0}^{\Delta T}\left(\mathrm{Qa}\left(t\right)\mathrm{Cr},a\left(t\right)-\mathrm{Qf}\left(t\right)\mathrm{Cr},f\left(t\right)\right)\mathrm{dt}=\Delta \mathrm{Vr}\gamma \mathrm{Cp}$$ (15)

The permeability fraction: γ is the integral in Eq. 15 divided by ΔVr Cp. Beyond the formula given by Yuan and Rigor (2011) on transvascular protein movement, more information on the quantities within the two integrals in Eqs 14, 15 are required for calculating the value of γ.

As an alternative, we used in our previous analysis the following reasoning to deduce 0.09 as the value for γ. First, we set over the pre-HD time, the protein concentration of the filtration to the tissue Cr,f in this form γ^1/2^ Cp. As the interstitial fluid space is being flushed by this fluid for a long time, the protein concentration of the interstitial fluid Ct can take Cr,f as its value. Over the course of HD, the endothelia may allow a similar fraction of protein to be restituted back to the circulation. Thus, we take the protein concentration of the restituted fluid as γ ^1/2^ Ct which is also γCp. On their study of the composition of interstitial fluid, Fogh-Andersen et al. (1995) reported that the PPC of their subjects in supine position is 6.86 g/dl and the protein concentration of interstitial fluid is 2.06 g/dl. These two protein concentrations lead us to set the value of γ ^1/2^ as 0.3 (≈2.06/6.86). This selection is equivalent to set γ as 0.09. As a sensitivity check, some computations on ΔVp/Vp are made later on with 0.4 as the value of γ.

**FIGURE 3:**
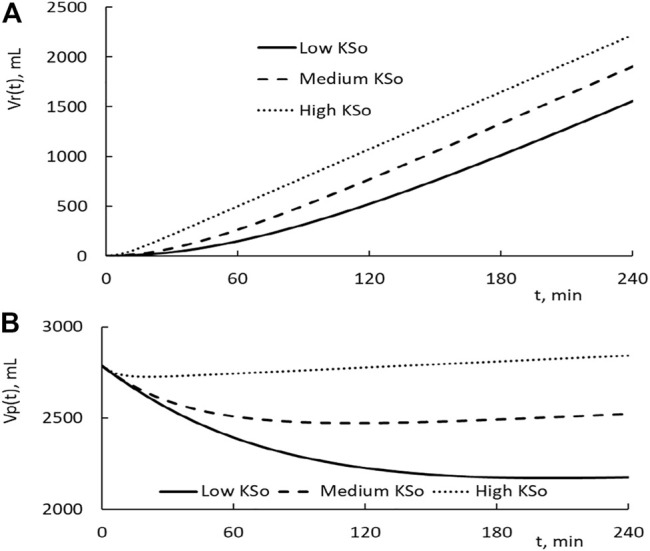
**(A)** Temporal changes in fluid restitution volume Vr(t) over the course of HD. The patients taking the HD have low, medium, and high filtration coefficient KSo. The plasma volume of the patient is set at 2,785 ml, and the HD is performed with the relative extraction ΔVe/Vp as 77.8%. **(B)** Temporal changes in plasma volume induced by HD.

*Dynamics of transvascular fluid and protein movement.* The time courses of the restitution volume Vr(t) for the HD with low, medium, and high KSo are depicted as the solid, broken, and dotted line, respectively, in Figure 3A. As one can see that the initial rise in restitution volume is much higher for patients with higher KSo. Over the later time, the rate of increase in Vr becomes comparable for the three HDs. Because of their difference over the early stage of HD, more fluid is restituted from the tissue for patients with higher KSo.

The subtraction of the extraction from the restitution is the reduction in plasma volume. Its time courses for the three HDs are depicted in Figure 3B. As the HD is initiated, the plasma volume is in a decreasing trend. Around the time of 24, 114, and 210 min, the plasma volume of the high, medium, and low KSo HD turns to an increasing trend, respectively. An increasing trend means that the rate of fluid restitution at that time is higher than the extraction rate. The decrease in Vp at the end of HD is larger for patients with lower KSo.

**FIGURE 4:**
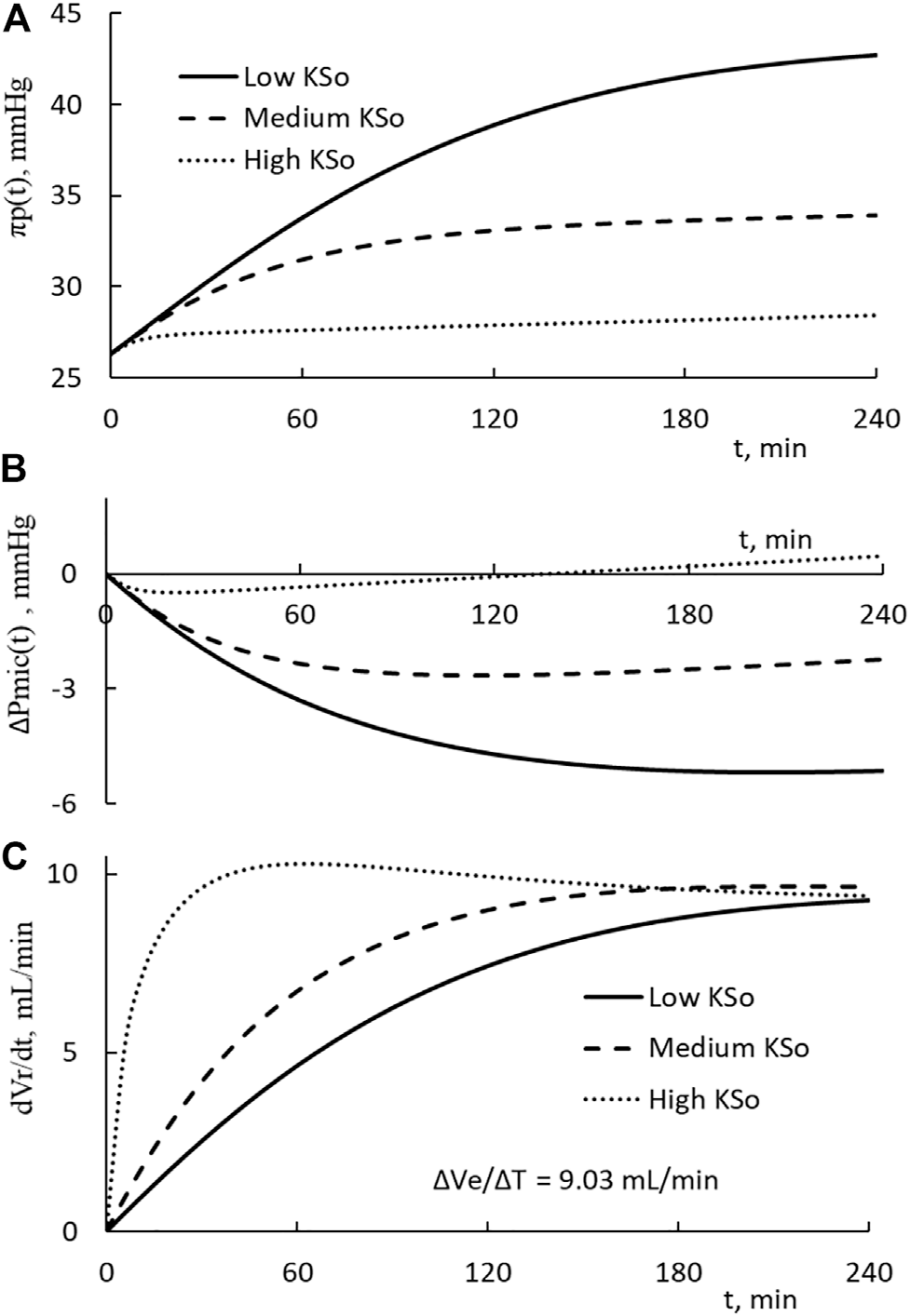
**(A)** Temporal increases on the plasma COP for patients with low, medium, and high filtration coefficient KSo. The HD is performed with the relative extraction ΔVe/Vp as 77.8%. **(B)** Temporal decreases in microvascular blood pressure. **(C)** Temporal changes of the rate of restitution.

The changes in plasma COP, microvascular blood pressure, and the rate of fluid restitution are shown in Figures 4A–C. The results on COP indicate that the PPC is in a monotonic increasing trend over the course of HD. The end COP is the largest for patients with the lowest KSo. The rate of restitution is shown to rise as the time progresses and then approaches asymptotically to about 9.3 ml/min which is slightly higher than the extraction rate of 9.03 ml/min.

The calculations presented in Figures 3, 4 are made with the same initial conditions of Vp, Vb, and Cp, the same modeling constant γ, ζ and the same HD parameters ΔVe and ΔT. The differences among the three HDs shown in Figures 3, 4 are the result that the filtration coefficient set for HD is different. However, there is one exception on the rate of restitution near the end of dialysis. At that time, the rates of the three HDs are about the same. Eq. 9b is made up of ΔVe/Vp, which describes how the extraction is to increase the PPC, and a term proportional to ΔVr(t)/Vp, which describes how the restitution is to dilute the PPC. Initially the increase in restitution volume is small and the restitution dilution is low. The high initial increment in PPC (ΔCp(t)/Cp’(t)) leads to a rapid increase in πp(t), Vr(t), and restitution dilution. Then, the increase in restitution dilution as indicated by Eq. 9b will slow down the rise in PPC. As the rate of restitution rises to the rate extraction, the PPC will increase no more. The plateauing of the restitution rate to the extraction rate, as shown in Figure 4C, is a condition projected by Eqs 5, 9b.

We can calculate the total driving pressure and the pressure fraction, ΔPmic(t)/[(Δπp(t)-ΔPmic(t)]. At 1 h, the fractions of low, medium, and high KSo HD take these percentages 31, 32, and 23.6%, respectively. The correspondent values at the end of HD are 23.9%, 22.6%, and -28.9%. These percentages indicate that the reduction in microvascular blood pressure contributes about 30% of the driving pressure to restitute the fluid from the tissue.

**FIGURE 5:**
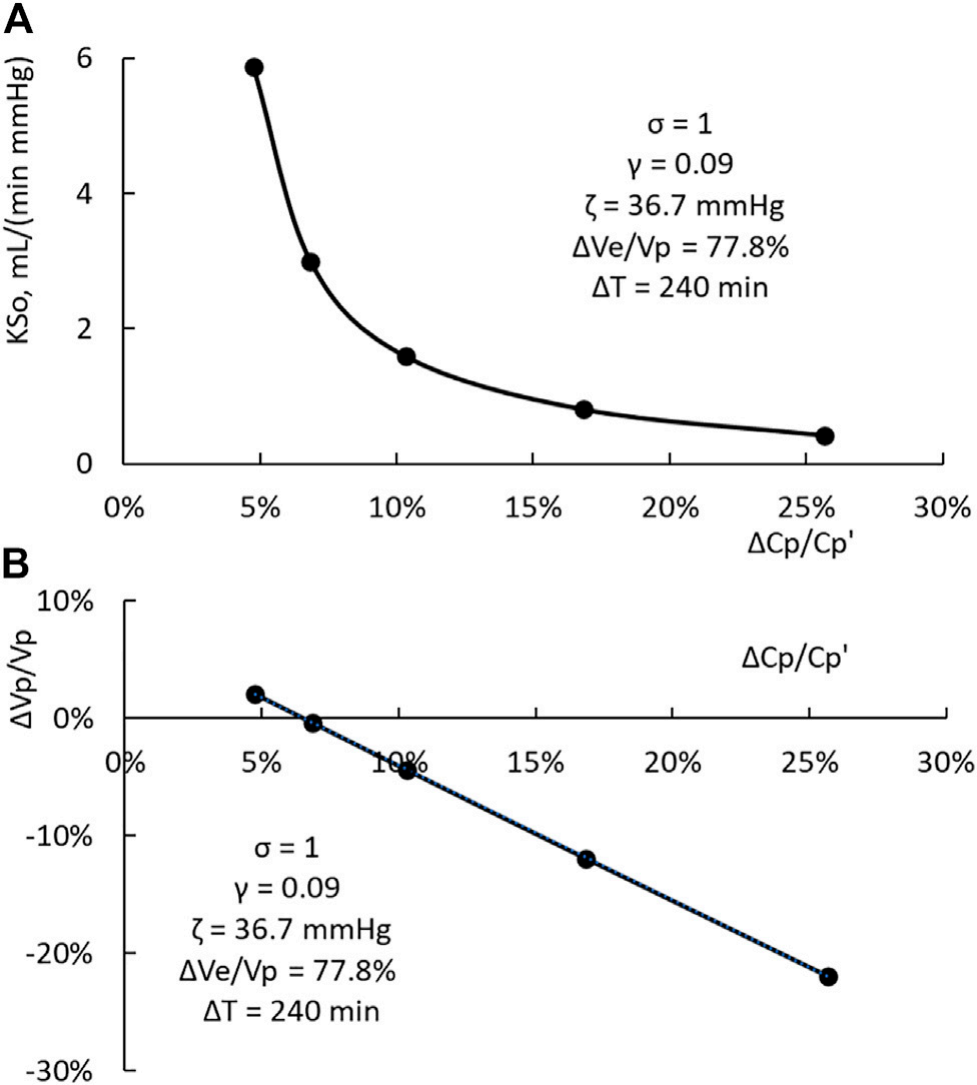
**(A)** One-to-one relation between KSo and the PPC increment ΔCp/Cp’. **(B)** One-to-one relation between the relative change in plasma volume ΔVp/Vp and the PPC increment.

For these three HDs, the filtration coefficient is the only variable being changed. The computation results indicate that KSo and ΔVp/Vp, as shown in Figure 5, have a one-to-one relation with the PPC increment ΔCp/Cp’. The data points in Figure 5A, computed from Eqs 8, 9b, can be matched by a straight line with a slope of 1.09 and a correlation coefficient (*R* ^2^) of 0.9996. If 0.4 is set as γ, the relation between ΔCp/Cp’ and ΔVp/Vp is slightly curvilinear. The slope of the straight line fit is 1.47 and the *R* ^2^ is 0.994. For a HD, ΔCp/Cp’ can be measured readily. If the HD is done with a ΔVe and ΔT similar to those used to derive the results shown in Figure 5, then the relations depicted in Figure 5A can be used to provide the first estimate of KSo and ΔVp/Vp.

**FIGURE 6:**
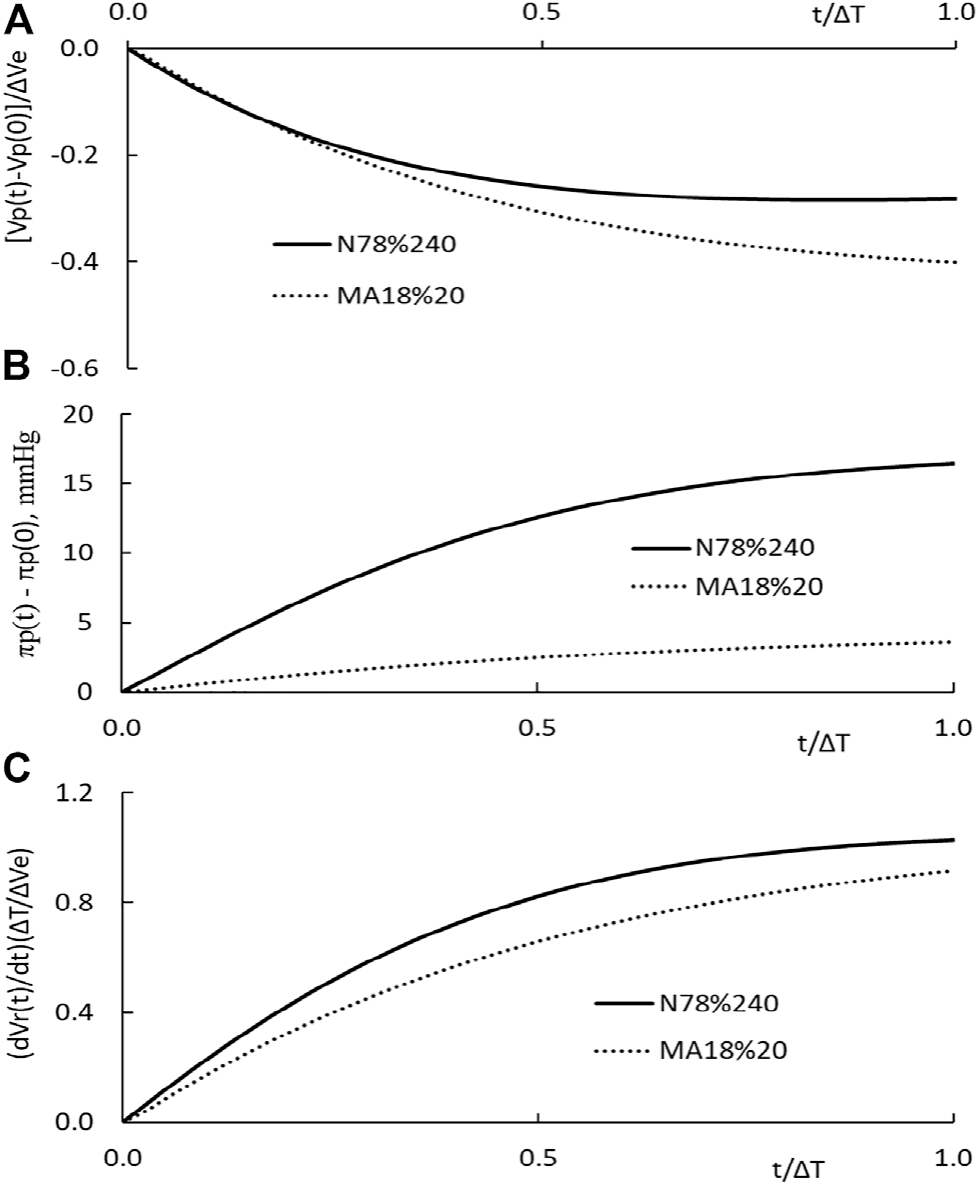
**(A)** Temporal changes in plasma volume of N78% HD carried out in patients with normal hematocrit (solid line) and that MA18%20 HD carried out in patients with more anemic hematocrit (dotted line). **(B)** Temporal changes in plasma COP πp(t) of the two HDs. **(C)** Temporal changes in the normalized rate of restitution dVr(t)/dt.

The time course of the plasma volume, PPC, and the restitution rate of N78% and MA18%20 HD is depicted in Figure 6. The HD with patients of normal hematocrit has ΔVe/Vp as 77.8%, and ΔT as 240 min. The ΔVe/Vp of the MA18%20 HD is 18.4%, and its ΔT as 20 min. The filtration coefficients used in the computations are 0.43 and 5.88 ml/min/mmHg, respectively. Their rate of extraction ΔVe/ΔT is 9.0 and 36.5 ml/min. To put these results for comparison, we normalize the time scale t of the figure by ΔT, the plasma volume change by ΔVe, and the rate of restitution by ΔVe/ΔT. A comparison of these results with those shown in Figure 3 indicates that a change in ΔVe and ΔT can significantly change the time courses of Vp(t) and dVr(t)/dt.

*Multiple circulation system.* Gibson et al. (1946) obtain tissue samples cleared of visible blood vessels and used the tagged RBC and plasma protein methodology to measure the blood volume of minute vessels in eight organs and the hematocrit of blood in minute vessels. The organ weight, total microvascular blood volume, and total blood volume of the eight organs are given in Table 2 The distribution of microcirculation volume Vmic,n and the ratio of the microvascular hematocrit Hmic,n to Ha of the eight organs are listed in the 6th column of Table 2. In reference to the weight of the *n*th organ (OWn), Vmic,n/OWn for these eight organs is in the range of 0.016–0.77 ml/g. We also calculate the normalized total blood volume of the *n*th organ (Vb,n)/OWn to highlight that the organ blood volume count is distributed over a wide range of 0.11–0.77 ml/g. The microvascular hematocrit of the spleen is 2.07 times of the arterial hematocrit. The other seven organs have a microvascular hematocrit in the range of 0.22–0.78 times of Ha. The volume weighted microvascular hematocrit for the seven organs (no spleen) is 0.47 Ha.

**TABLE 2:** Organ weights, volumes of minute vessels, microvascular hematocrit, and the organ KSn product of a normal dog^a^.

	OWn, g	Vmic,n, mL	Vmic,n/OWn mL/g	Vb,n, mL	Hmic,n/Ha	KSn/OWn	KSn
Spleen	72	36.7	0.510	47.5	2.07		
Liver	99	37.1	0.375	76.2	0.31	0.80	1.15
Lungs	252	29.9	0.119	65.2	0.78	4.55	0.15
Kidneys	58	4.7	0.081	25.7	0.21		
Heart	83	5.6	0.067	11.2	0.52		
Bowel	350	14.4	0.041	38.3	0.36		
Muscle	3540	57.1	0.016	130.4	0.45	0.14	0.50
Brain	62	2.0	0.032	6.6	0.21		

a The data in the 2nd to 6th column are derived from Table 1 of Gibson et al. (1946). OWn stands for organ weight. The total blood volume Vb of the dog is 1,150 ml, and the dog weighs 11.9 kg. The units of KSn/OWn and KSn are mL/(min mmHg kg) and mL/min/mmHg, respectively.

Measured by the isogravimetric technique, the KSn/OWn of the liver (Bennett and Rothe 1981), lung lobe (Lee et al., 1996), and hindlimb (identified as the muscle, Michel 1984) are listed in the 8th column of Table 2. In view of the alveolar structure of the lung and the dense vasculature of the liver, the endothelial surface area of the lungs per unit lung weight (Sn/OWn) may be the largest of the three organs and that of the muscle is the smallest. The value of KSn/Own and KSn are listed in the last two columns of Table 2.

The circulatory system is consisted of the vascular trees of various organs. From the perspective of the circulatory system, these trees can be viewed as multiple circulations arranged in parallel. Let the filtration coefficient of the *n*th circulation be KSn and its plasma volume be Vp,n. Then, we set the filtration coefficient KSo and the total plasma volume Vp of the circulatory system at:

$$\mathrm{KSo}=\sum_{n=1}^{m}\mathrm{Ksn}$$ (16a)

$$\mathrm{Vp}=\sum_{n=1}^{m}\mathrm{Vp},n$$ (16b)

For the given ΔVe and Cp, we can use the values of KSo and Vp so determined to calculate Cp’, ΔCp/Cp’, ΔVr, ΔVp, ΔPmic, and Δπp .

Let us define the KS fraction λn of the *n*th circulation as:

λn=KSn/Kso (17)

and assign λn ΔVe as the extraction imposed to the *n*th circulation, λnVp as its plasma volume, and λnVb as its blood volume. λn ΔVr will be set at the fluid volume restituted from the *n*th circulation. The substitution of these parameters into Eqs 5–10 will reveal that all λn values are canceled out, and the five equations remain in the same forms. Remaining in the same form means that the values of Cp’, ΔCp/Cp’, ΔPmic, and Δπp calculated for the *n*th circulation will be identical to those derived for the multiple circulation system. This identity justifies the use of Eq. 16a to derive the KSo of the entire circulatory system. The limited data on KSn shown in Table 2 suggest that a significant fraction of the fluid restituted from the tissue through HD would be derived from the liver.

The plasma volume Vp,n set for the *n*th organ with the λ_n_ in Eq. 17 may not be the true plasma volume of that organ. Thus, the PPC of the plasma coming out from the *n*th organ will be different from the one calculated for the multiple system. As the plasma is being mixed in the heart chambers, the mixed final PPC becomes the one predicted by the FCA for the multiple system.

The FCA of PPC increment induced by the HD of more anemic patients indicates that the value of KSo/BW is 0.11 ml/(min mmHg kg). If we apply this value to a dog of 11.9 Kg, then its KSo will be 1.33 ml/(min mmHg), which is smaller than the sum of the three values given in the last column of Table. 2.

*Microvessel hematocrit, whole body hematocrit, and Fcell ratio.* In FCA, the circulation (not including the splenic microcirculation) is divided into a microcirculation and a macrocirculation compartment. Their blood volumes are designated as Vmic and Vmac. The hematocrit of the blood in these two compartments are Hmic and Ha. We use the fractional numbers α and *β* to describe the following relations:

$$\begin{array}{ccc} \mathrm{Hmic}=\alpha \mathrm{Ha} & \mathrm{Vmic}=\beta \mathrm{Vb} & \mathrm{Vmac}=\left(1-\beta \right)\mathrm{Vb} \end{array}$$ (18)

The whole blood hematocrit Hw, the total RBC volume of the two compartments divided by the blood volume, and the Fcell ratio (=Hw/Ha) of the two compartment circulation can be calculated as:

Hw=(Ha Vmac+Hmic Vmic)/Vb=(1−β)Ha+αβ Ha (19a)

Fcell≡Hw/Ha=1−β+αβ (19b)

Eq. 19a indicates that Hw is a volume weighted average hematocrit of the two compartments. We can form a full circulation by adding the pulmonary circulation and the four heart chambers to the central circulation shown in Table 1. The sum of the microvessel blood volumes in this full circulation yields a value of *β* close to 0.326. The volume weighted hematocrit of the full circulation yields a value of α close to 0.54. The selection of 0.326 and 0.54 will make the Fcell defined in Eq. 19b to take 0.85 as its value. If we set the volume of splenic microcirculation that contains high hematocrit blood as 4.2% of Vb and its hematocrit as 2.07 Ha, the Fcell for the micro- and macrocirculation and the splenic microcirculation will be increased to 0.90.

For comparison, Dasselaar et al. (2007) reported that the Fcell ratio measured for their HD patients is 0.896 ± 0.036. In their article, Chien and Gregersen (1962) point out that Fcell of subjects is 0.9 and that of subjects with no spleen is 0.85.

The initial value of *β* is 0.326. As the time progresses, the microvascular blood volume and the blood volume will be decreased by the HD. As calculated for N78% HD, the value of *β* derived from the FCA is reduced to 0.273 and the Fcell ratio is increased to 0.874. For calculations, we assume that the fractional constant α is not altered by the change in microvascular blood volume.

*Factors changing the arterial hematocrit.* A change in plasma volume ΔVp, a change in microvascular blood volume ΔVmic, and a shift of the concentrated RBC from the spleen ΔVrbc to the circulation lead to the following change in the arterial hematocrit of blood circulating through the macrocirculation:

ΔHa/Ha′=[−ΔVp/Vb+(1−α)ΔVmic/Vb+(ΔVrbc/Vb)/Ha′]/Fcell (20)

This equation is derived from the conservation of RBC in the macrocirculation compartment and is termed as the hematocrit equation.

The results of CHRE indicate that the reduction in microcirculation blood volume due to hemorrhage is linearly related to the reduction in blood volume by the following equation:

ΔVmic=ηΔVb (21)

The constant η takes 0.65 as its value (LaForte et al., 1994). In view of the large volume distributions in the small veins (Table 1), most of the ΔVmic could be originated from the volume reduction of small veins (the 10th generation).

In our previous analysis on the data of A72% and Ma18%20, if ΔVrbc is set as zero, the FCA projects that the microcirculation would be dilated by 3.3%Vb and 2.5%Vb, respectively. The arterioles are the only microvessel generation that could be induced by HD to dilate. As shown in Table 1, the arterioles contain only 1.2% of the blood volume. The large dilatation percentages estimated for ΔVmic/Vb indicate that the spleen is activated by the HD to release the concentrated RBC at these volumes (ΔVrbc*): 0.03%Vb (N78%), 1.09%Vb (A78%), and 0.91%Vb (MA18%). These percentages are derived with η set as 0.65. If we decrease η to 0.4 (i.e., the microcirculation is more rigid), the FCA projects that the spleen of patients of normal hematocrit is induced by N78% HD to retain 0.74%Vb of the concentrated RBC from the general circulation. The reduction in total radioactivity of the tagged RBC in the spleen found by Yu et al. suggests that the spleen is not likely to be activated by the HD to retain RBC from the central circulation.

To illustrate how splenic RBC release and microvascular volume reduction change arterial hematocrit over the course of HD, we set ΔVp/Vp as the one derived from Eq. 7, ΔVmic as the one derived from Eq. 21 with η set at 0.65, and ΔVrbc(t) computed from the following linear function of t:

$$\Delta \mathrm{Vrbc}\left(t\right)=\Delta \mathrm{Vrb}c^{*}\cdot \frac{t}{\Delta T}$$ (22)

**FIGURE 7:**
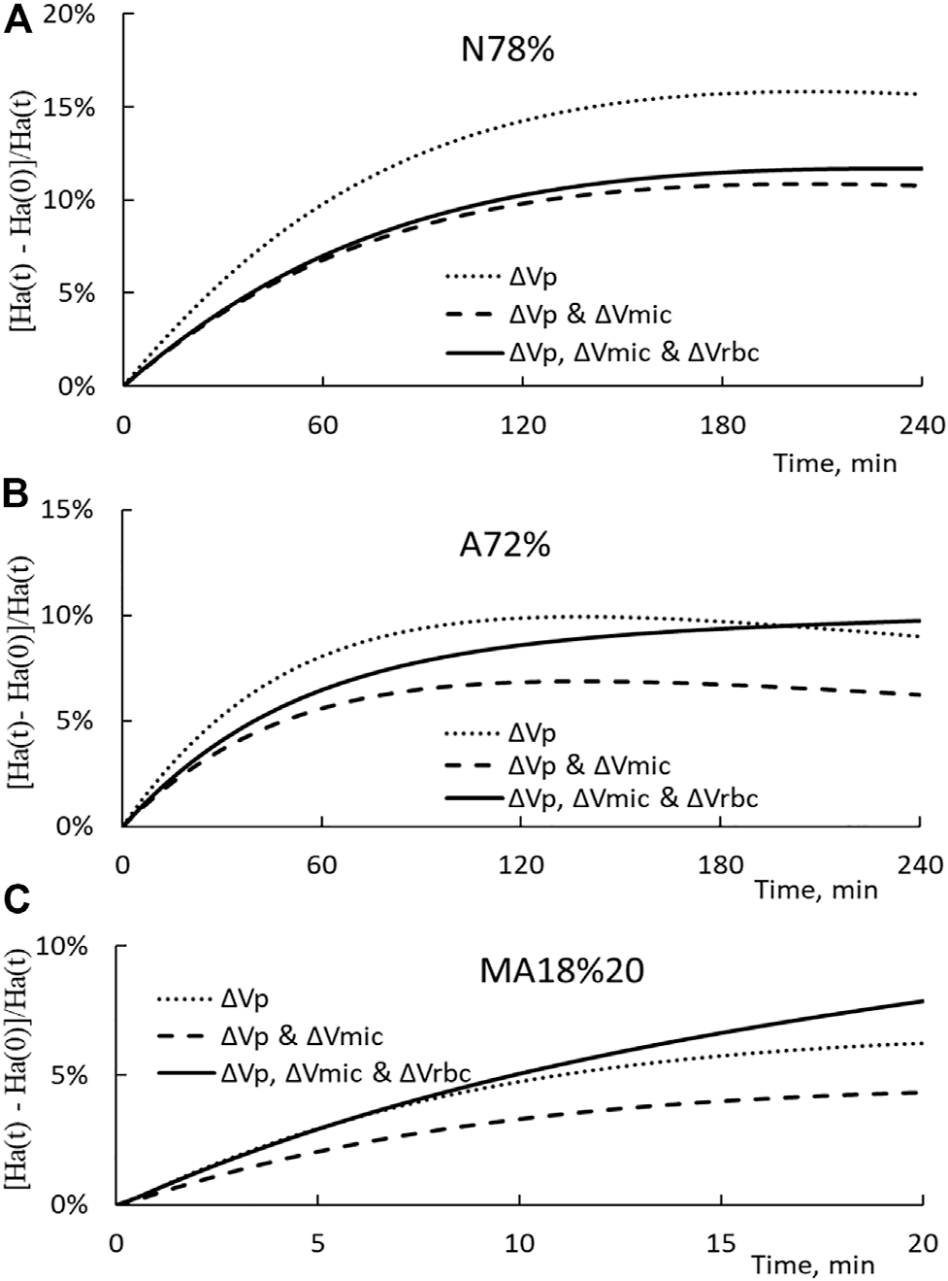
**(A)** Temporal changes in the arterial hematocrit increment Ha(t)-Ha (0)/Ha(t) for the N78% HD. The dotted, broken, and solid lines show the increment induced only by ΔVp, ΔVp, and ΔVmic, and all three ΔVp, ΔVmic, and ΔVrbc, respectively. **(B)** Temporal changes in the arterial hematocrit increment for A72% HD. **(C)** Temporal changes in the arterial hematocrit increment for MA18%20 HD.

The arterial hematocrit Ha(t) is calculated first with ΔVp only (the dotted line), then ΔVp and ΔVmic (the broken line), and finally with ΔVp, ΔVmic, and ΔVrbc (the solid line). The hematocrit changes for N78%, A72%, and MA18%20 HD are presented in Figures 7A–C respectively. It is noted that the end point of the solid line corresponds to the hematocrit Ha’ measured in the experiments. The results shown in the figure indicate that the reduction in the microvascular blood volume will reduce the hematocrit from that induced by the plasma volume change. In the opposite direction and as expected, splenic RBC releases will lead to an increase in the arterial hematocrit.

If HD does not induce the spleen to release its highly concentrated blood to the circulation (i.e. ΔVrbc = 0) and the microcirculation is rigid so that ΔVmic = 0, then Eq. 20 is simplified to the following form:

ΔHa/Ha′=−ΔVb/Vb/Fcell (23)

If we set Fcell at unity and replace ΔVp by ΔVb (Eq. 8), then Eq. 23 is further simplified to VBHE, which is widely used to determine the relative change in the blood volume (RCBV and ΔVb/Vb) induced by HD. For the N78%, A72%, and MA18%20 HD, the VBHE projects that the patients would have RCBV as -11.7%, -10.2%, and -7.9%, respectively. The correspondent changes derived from the previous FCA are -14.5%, -8.3%, and -5.6%.

The RCBV measured by a tagging technology is assessed as −17.3% and that calculated by VBHE is −8.2% (Dasselaar et al., 2007). They conclude that the VBHE underestimates RCBV for patients with normal hematocrit and overestimates for patients with anemic hematocrit. The hematocrit of their patients is about 40% which is slightly below the normal hematocrit (42.4%) referred in thisarticle. Their conclusion is compatible to our finding that the RCBV derived from the VBHE for patients with normal hematocrit is smaller than the RCBV derived from the FCA.

*Reduction in macrovascular blood volume.* Since the sum of macro- and microvascular blood volume is the total blood volume, we have the change in macrovascular blood volume as:

ΔVmac=ΔVb−ΔVmic (24)

In view of the large volume fractions of the venous macrocirculation (Table 1), most of the ΔVmac could be originated from the venous macrocirculation. For N78%, A72%, and MA18% HD, the FCA projects the macrovascular volume reduction values (ΔVmac/Vb) are −4.9%, −2.9%, and −2.0, respectively.

The venous macrocirculation has been regarded as a volume reservoir that can be used to improve venous return and cardiac filling. Because the right atrium resides downstream of the venous macrocirculation, it is likely that the macrovascular blood volume reduction derived from the FCA can be used as an index in characterizing whether the cardiac filling is being reduced by the HD.

*Comparisons with other experimentations and analyses.* LaForte et al. (1994) carried out their hemorrhage (5, 10, and 15% of the blood volume) in 2 min and then reinfusion in the next 2 minutes. Over this short period, the volume of the fluid restituted from the tissue will be much smaller than that of HD performed over 4 h. Similar changes in the hematocrit are also found for rabbits with their spleen removed (LaForte et al., 1992). Thus, the hematocrit decreases over the 2-min hemorrhage is generated mostly by a reduction in the microvascular volume. For 10% hemorrhage experiments, they found that the PPC is reduced by 0.027 ± 0.008 g/dl and the hematocrit by 1.20 ± 0.04%. The correspondent PPC increment ΔCp/Cp’ is 0.5%, and the hematocrit increment ΔHa/Ha’ is 3.6%. The analysis of the protein and hematocrit data yields these estimations:

(i) The splenic RBC release is minimally induced by CHRE, and the rabbit’s microcirculation functions like a passive elastic system.
(ii) The hemorrhage volume of 7% is derived from the fluid being restituted from the tissue over 2 min, 60% from the reduction in microvascular blood volume (= ΔVmic), and 33% from the reduction in macrocirculation blood volume (= ΔVmac),
(iii) The filtration coefficient of rabbit as projected by FCA is 0.21 ml/(min mmHg kg). The correspondent estimate derived from FCA of three HDs has the filtration coefficient in the range of 0.008–0.085 ml/(min mmHg kg).
(iv) If the VBHE is used to compute ΔVb and Eq. 7 to calculate ΔVr, then the calculation by Eq. 5 will yield 1.08 ml/(min mmHg kg) as the filtration coefficient of the rabbit, which is almost 5 times larger than the one estimated by the FCA.

For the HDs analyzed here, the dialysis is done over a long period of time, the fluid volume restituted from the tissue can be significantly larger than that generated from the CHRE of rabbits. As a result, the PPC and hematocrit increments found for the three HDs analyzed here have comparable values. The difference between these two increments indicates that the HD does induce significant change in microvascular blood volume and cause the spleen to release RBC to the circulation.

On the MA18%20 HD, Schneditz et al. (1992) used a protein analysis to derive the RCBV. Then the matching of the RCBV with that derived from the hematocrit data leads to a projection that the filtration coefficient of the more anemic patients is 5.6 ml/(min mmHg 50 Kg). This is equivalent to set the value of KSo as 7.74 ml/min/mmHg (= 69.1 Kg (BW) Χ 5.6 ml/(min mmHg 50 Kg)). In our FCA, we match the projected Cp’ with the measured one and deduced KSo as 5.9 ml/min/mmHg. The difference in data matching (hematocrit vs PPC) may be the reason that the estimate of Schneditz et al. (1992) on KSo is different from our estimate.

## 3 Concluding Remarks

In this article, we use the FCA to determine from the protein data of the three HD studies, namely, the restitution volume, the change in plasma volume, and the filtration coefficient of patients with normal, anemic, and more anemic hematocrit. To further verify FCA and the modeling of the circulation, we recommend that the following experiments be performed:

(i) The making of hourly PPC measurements over the course of HD. The measurements will verify whether the FCA projected the temporal changes in PPC correctly.
(ii) A total of two HDs with two different relative extractions (i.e., at two ΔVe/Vp) deriving the filtration coefficients from the protein data of these two HDs should have the same value. In cases that they do not, a different guess on the filtration coefficient and permeability fraction may lead FCA to generate results that can match the two measured PPC increments.
(iii) The performance of the tagged protein and RBC experiments of Dasselaar et al. (2007) and the use of their procedure projected the reduction in plasma volume. With the PPC also measured in these experiments, then we can examine whether the reduction in plasma volume projected by FCA matches that by the tagging technology.
(iv) The performance of the CHRE for the determination of η so that Eq. 20 can be used to determine the splenic RBC release.
(v) Yu et al. (1997) found that the splanchnic radioactivity decreases to 90% of the baseline value after 2 h of the accelerated fluid removal during dialysis. The volume reduction of the splanchnic macrocirculation can cause a decrease in radioactivity. If this radioactive technology can be shown to measure the splenic RBC release, then we have an independent way to verify the splenic RBC release projected by FCA.

The analysis of HD data with patients grouped according to their hematocrits leads to the recognition that patients having higher hematocrits are to have a smaller filtration coefficient and the suggestion that the filtration coefficient is a facilitator to generate more fluid restitution. As a result, we have a better compensation to the fluid extraction by the dialyzer and a lesser reduction in plasma volume for patients with lower hematocrit. It will be of interest to group patients according to their frequency of having intradialytic hypotension and/or their blood pressure variability (Flythe and Brunelli 2014) so that we can better understand what other factors can also be a limiting factor on having more fluid restitution. Through these experiments and data comparison, a better understanding of the factors inducing hypovolemia for patients taking HD can be the basis for one to modify the HD process such that the development of hypovolemia can be avoided over the course of HD.

## Data Availability

The raw data supporting the conclusion of this article will be made available by the authors, without undue reservation.

## 4 Appendix: Equations and Computation Procedure

The entire HD period is divided into M segments. For each segment [i∙dt to (i + 1)dt], the volume of fluid extracted by the dialyzer is dVe (= ΔVe/M). The initial value Cp(0), Vp(0), and Vb(0) are correspondent to the one set for Cp, Vp, and Vb in Eqs 3–5. In addition, we also have Vr (0) as zero. To start the calculations, we will set a value as KSo. Then, we guess the PPC increment for the *i*th segment as dCp. The values of various variables are computed by the following equations:

Cp(i+1)=Cp(i)+dCp
(A1)

Vr(i+1)=Vr(i)+[dVe−dCp Vp(0)/Cp(i+1)]/(1−γCp(0)/Cp(i+1))
(A2)

Vp(i+1)=Vp(i)+Vr(i+1)−Vr(i)−dVe
(A3)

Vb(i+1)=Vb(i)+Vp(i+1)−Vp(i)
(A4)

Pmic(i+1)=Pmic(0)+ζ[Vb(i+1)−Vb(0)]/Vb(0)
(A5)

$$\pi p\left(i+1\right)=2.1\mathrm{Cp}\left(i+1\right)+0.16\mathrm{Cp}{\left(i+1\right)}^{2}+0.009\mathrm{Cp}{\left(i+1\right)}^{3}$$
(A6)

KS1=2[Vr(i+1)−Vr(i)]/dt/{σ[πp(i+1)+πp(i)−2πp(0)]−Pmic(i+1)−Pmic(i)+2Pmic(0)}
(A7)

The value of dCp is changed until the value of KS1 takes the value of KSo. Then, similar computations are performed for the next segment. The value of Cp(M), Vr(M), and Vp(M)-Vp(0) is taken as Cp’, ΔVr, and ΔVp, respectively. The value of k in Eq. 4 is computed as

$$\left(\sigma \Delta \pi p-\Delta \mathrm{Pmic}\right)k=\left[\int_{0}^{\Delta T}\left\{\sigma \pi p\left(t\right)-\sigma \pi p\left(0\right)-\mathrm{Pmic}\left(t\right)+\mathrm{Pmic}\left(0\right)\right\}\mathrm{dt}\right]$$
(A8)

Experimentally, the value of Cp’ is reported. Then, we can use Figure 5 to set a likely value for KSo. Iterations will be performed until the resulting Cp’ takes the experimentally measured value. The final value found for KSi is taken as KSo of that patient.

## Glossary

(t): Quantity without ’ is that at beginning time 0 and with ’ that at the end time ΔT. For temporal calculations, (t) is added to the quantity.
BW: Body weight in kg.
CHRE: Cyclic hemorrhage and reinfusion experiment.
COP: Colloidal osmotic pressure.
Cr: The protein concentration in fluid restituted from the tissue to the circulation, Eqs 9, 10.
FCA: Full circulation analysis. The full circulation is represented by a macrocirculation, a microcirculation, and a splenic microcirculation.
Fcell: The ratio of the whole body hematocrit to the arterial hematocrit, Eq. 19b.
HD: Hemodialysis.
Hw: Whole body hematocrit, Eq.19a.
k: Constant in Eq. 5 and Eq. A8.
KSo: Filtration coefficient with So representing the surface available for filtration
Pa: Arterial blood pressure.
Pb: Base pressure which is πp–πt–Pt. See Eq. 11.
Pmic: Microvascular blood pressure. See Eqs. 2, 11, 12.
PPC: Plasma protein concentration.
Pv: Venous blood pressure.
RBC: Red blood cell.
RCBV: Relative change in blood volume.
Rn: Flow resistance of the *n*th generation
S-axis: Summation of the surface areas upstream of the point in the vasculature divided by So.
Vb: Blood volume which is the sum of Vmac and Vmic.
VBHE: van Beaumont hematocrit equation, Eq. 23 with Fcell set at 1.
Vmac: Macrocirculation volume = (1-*β*) Vb.
Vmic: Microcirculation volume = *β* Vb.
Vp: Plasma volume = (1-Hw) Vb.
Vr: Restitution volume.
α: = Hmic/Ha.
β: = Vmic/Vb. The value of *β* can change over the course of HD.
γ: Permeability fraction, Eq. 10. γCp is taken as the protein concentration Cr in the fluid being restituted from the tissue to the circulation.
ΔPmic: Change in microvascular blood pressure = Pmic’-Pmic. Eqs 5, 6.
ΔT: HD time.
ΔVe: Extraction volume or volume of ultrafiltrate being extracted by the dialyzer.
ΔVp: Change in plasma volume = Vp’-Vp.
ΔVr: Restitution volume or the total volume of the fluid restituted from the tissue.
Δπp: Change in plasma COP = πp’-πp. Eq. 5.
ζ: Autonomous constant which defines how the autonomous control changes the relation between ΔPmic and ΔVb. Eq. 6
η: Fraction constant relating the change in microvascular blood volume to the change in total blood volume, See Eq. 21.
λ_n_: = KSn/KSo, where KSn is the filtration coefficient of the *n*th organ and KSo is that of the microcirculation. Eq. 17.
πp: Plasma COP.
πt: Tissue COP.
σ: Reflection coefficient, Eq. 2.

## References

[B1] Bennett T. D. Rothe C. F. (1981). Hepatic Capacitance Responses to Changes in Flow and Hepatic Venous Pressure in Dogs. Am. J. Physiol. 240, HI8–H28. 10.1152/ajpheart.1981.240.1.H18 7457619

[B2] Booth J. Pinney J. Davenport A. (2011). Do Changes in Relative Blood Volume Monitoring Correlate to Hemodialysis-Associated Hypotension. Nephron Clin. Pract. 117, c179–c183. 10.1159/000320196 20805690

[B3] Cavalcanti S. Cavani S. Ciandrini A. Avanzolini G. (2006). Mathematical Modeling of Arterial Pressure Response to Hemodialysis-Induced Hypovolemia. Comput. Biol. Med. 36, 128–144. 10.1016/j.compbiomed.2004.08.004 16389073

[B4] Chien S. Gregersen M. I. (1962). “Determination of Body Fluid Volumes,” in Physical Techniques in Biological Research. Editor Nastuk W. L. (Cambridge, Massachusetts, United States: Academic Press), 1–105. Chapter 1. 10.1016/b978-1-4831-6742-8.50008-2

[B5] Dasselaar J. J. Lub-de Hooge M. N. Pruim J. Nijnuis H. Wiersum A. de Jong P. E. (2007). Relative Blood Volume Changes Underestimate Total Blood Volume Changes during Hemodialysis. Cjasn 2, 669–674. 10.2215/CJN.00880207 17699480

[B6] Flythe J. E. Brunelli S. M. (2014). Blood Pressure Variability and Dialysis: Variability May Not Always Be the Spice of Life. J. Am. Soc. Nephrol. 25, 650–653. 10.1681/ASN.2013111237 24385594 PMC3968509

[B7] Fogh-Andersen N. Altura B. M. Altura B. T. Siggaard-Andersen O. (1995). Composition of Interstitial Fluid. Clin. Chem. 41, 1522–1525. 10.1093/clinchem/41.10.1522 7586528

[B8] Fung Y. C. (1966). Theoretical Considerations of the Elasticity of Red Cells and Small Blood Vessels. Fed. Proc. 25, 1761–1772. 5927411

[B9] Gibson J. G. II Seligman A. M. Peacock W. C. Aub J. C. Fine J. Evans R. D. (1946). The Distribution of Red Cells and Plasma in Large and Minute Vessels of the Normal Dog, Determined by Radioactive Isotopes of Iron and Iodine 1. J. Clin. Invest. 25, 848–857. 10.1172/jci101772 PMC435630 16695382

[B10] LaForte A. J. Lee L. P. Rich G. F. Skalak T. C. Lee J. S. (1994). Blood Volume Redistribution from a Passive Elastic Permeable Microcirculation Due to Hypovolemia. Am. J. Physiology-Heart Circulatory Physiol. 266, H2268–H2278. 10.1152/ajpheart.1994.266.6.h2268 7912898

[B11] LaForte A. J. Lee L. P. Rich G. F. Skalak T. C. Lee J. S. (1992). Fluid Restitution and Shift of Blood Volume in Anesthetized Rabbits Subject to Cyclic Hemorrhage. Am. J. Physiology-Heart Circulatory Physiol. 262, H190–H199. 10.1152/ajpheart.1992.262.1.h190 1346357

[B12] Landis E. M. Pappenheimer J. R. (1963). “Exchange of Substances through the Capillary walls,” in Handbook of Physiology, Section 2, Circulation (Vol II). Editors Hamilton W. F. Dow P. Washington D. C. (Rockville, Md: APS), 961–034.

[B13] Lee J. S. Lee L. P. Rothe C. F. (1996). Assessing Microvascular Volume Change and Filtration from Venous Hematocrit Variation of Canine Liver and Lung. Ann. Biomed. Eng. 24, 25–36. 10.1007/BF02770992 8669715

[B14] Lipowsky H. H. Usami S. Chien S. (1980). *In Vivo* measurements of "apparent Viscosity" and Microvessel Hematocrit in the Mesentery of the Cat. Microvasc. Res. 19, 297–319. 10.1016/0026-2862(80)90050-3 7382851

[B15] Michel C. C. (1984). “Fluid Movement through Capillary walls,” in Handbook of Physiology, Cardiovascular System, Microcirculation. Editors Pries A. R. Secomb P. W. (Rockville, Md: APS), 375–409. Chap. 9.

[B16] Minutolo R. Nicola L. D. Bellizzi V. Iodice C. Rubino R. Aucella F. (2003). Intra- and post-dialytic Changes of Haemoglobin Concentrations in Non-anaemic Haemodialysis Patients. Nephrol. Dial. Transplant. 18, 2606–2612. 10.1093/ndt/gfg387 14605285

[B17] Rothe C. F. (2011). “Venous System: Physiology of the Capacitance Vessels,” in Handbook of Physiology - Cardiovascular System. Editors Bohr D. F. Somlyo A. P. SparksJr. H. V. (Oxford, United Kingdom: Oxford University Press), 397–452. Chapter 13. 10.1002/cphy.cp020313

[B18] Schneditz D. Roob J. Oswald M. Pogglitsch H. Moser M. Kenner T. (1992). Nature and Rate of Vascular Refilling during Hemodialysis and Ultrafiltration. Kidney Int. 42, 1425–1433. 10.1038/ki.1992.437 1474776

[B19] Van Beaumont W. (1972). Evaluation of Hemoconcentration from Hematocrit Measurements. J. Appl. Physiol. 32, 712–713. 10.1152/jappl.1972.32.5.712 5038863

[B20] Yu A. W. Nawab Z. M. Barnes W. E. Lai K. N. Ing T. S. Daugirdas J. T. (1997). Splanchnic Erythrocyte Content Decreases during Hemodialysis: A New Compensatory Mechanism for Hypovolemia. Kidney Int. 51, 1986–1990. 10.1038/ki.1997.270 9186892

[B21] Yuan S. Y. Rigor R. R. (2011). “Regulation of Endothelial Barrier Function,” in Colloquium Series on Integrated Systems Physiology: From Molecule to Function. Editors Granger D. N. Granger J. P. (San Rafael CA: Morgan & Claypool Life Sciences), 1–146. Chapter 1. 10.4199/c00025ed1v01y201101isp013 21634066

